# Sleeping Beauty transposon system for GDNF overexpression of entrapped stem cells in fibrin hydrogel in a rat model of Parkinson’s disease

**DOI:** 10.1007/s13346-023-01289-9

**Published:** 2023-02-28

**Authors:** Laura Stahn, Justyna Rasińska, Tilo Dehne, Stefanie Schreyer, Aileen Hakus, Manfred Gossen, Barbara Steiner, Shabnam Hemmati-Sadeghi

**Affiliations:** 1 Department of Neurology, Charité – Universitätsmedizin Berlin, Corporate Member of Freie Universität Berlin, Humboldt-Universität Zu Berlin, and Berlin Institute of Health, 10115 Berlin, Germany; 2 Tissue Engineering Laboratory, Berlin-Brandenburg Center for Regenerative Therapies, Department of Rheumatology & Clinical Immunology, Charité – Universitätsmedizin Berlin, Charitéplatz 1, 10117 Berlin, Germany; 3 BIH Center for Regenerative Therapies (BCRT), Berlin Institute of Health at Charité-Universitätsmedizin Berlin, Charitéplatz 1, 10117 Berlin, Germany; 4 Institute of Active Polymers, Helmholtz-Zentrum Hereon, 21502 Teltow, Germany

**Keywords:** Neurodegeneration, Adipose tissue–derived mesenchymal stromal cells (adMSCs), Neurotrophic factors, Fibrin hydrogel, Transplantation, Tissue engineering

## Abstract

There is currently no causal treatment available for Parkinson’s disease (PD). However, the use of glial cell line–derived neurotrophic factor (GDNF) to provide regenerative effects for neurons is promising. Such approaches require translational delivery systems that are functional in diseased tissue. To do so, we used a non-viral Sleeping Beauty (SB) transposon system to overexpress GDNF in adipose tissue–derived mesenchymal stromal cells (adMSCs). Entrapment of cells in fibrin hydrogel was used to boost potential neurorestorative effects. Functional GDNF-adMSCs were able to secrete 1066.8 ± 169.4 ng GDNF/120,000 cells in vitro. The GDNF-adMSCs were detectable for up to 1 month after transplantation in a mild 6-hydroxydopamine (6-OHDA) hemiparkinson male rat model. Entrapment of GDNF-adMSCs enabled GDNF secretion in surrounding tissue in a more concentrated manner, also tending to prolong GDNF secretion relatively. GDNF-adMSCs entrapped in hydrogel also led to positive immunomodulatory effects via an 83% reduction of regional IL-1β levels compared to the non-entrapped GDNF-adMSC group after 1 month. Furthermore, GDNF-adMSC-treated groups showed higher recovery of tyrosine hydroxylase (TH)-expressing cells, indicating a neuroprotective function, although this was not strong enough to show significant improvement in motor performance. Our findings establish a promising GDNF treatment system in a PD model. Entrapment of GDNF-adMSCs mediated positive immunomodulatory effects. Although the durability of the hydrogel needs to be extended to unlock its full potential for motor improvements, the neuroprotective effects of GDNF were evident and safe. Further motor behavioral tests and other disease models are necessary to evaluate this treatment option adequately.

**Graphical Abstract:**

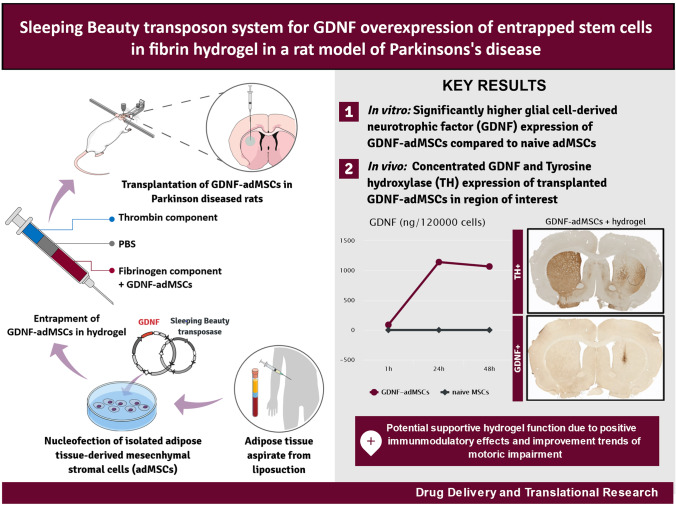

**Supplementary Information:**

The online version contains supplementary material available at 10.1007/s13346-023-01289-9.

## Introduction

Over the past 20 years, the global burden of Parkinson’s disease (PD) has more than doubled, reaching 6.1 million diseased individuals in 2016. At present, it is the fastest growing neurological disorder in terms of death, prevalence, and disability [1–3]. There is still no known primary cause for the pathological hallmark of PD, namely the selective degeneration of the nigrostriatal dopaminergic pathway with progressive loss of dopaminergic neurons in the *substantia nigra pars compacta* (SNpc) [4]. The characterization of early stages is still ongoing, although substantial progress has been made in the development of prodromal biomarkers [5–7]. Clinically, it is characterized not only by major motor symptoms like resting tremor, bradykinesia, rigidity, and postural instability, but also by a variety of other symptoms such as diminished smell, depression, and later, cognitive decline. Currently, diagnosis is primarily based on clinical manifestation. This results in symptomatic therapies only focusing on treating motor symptoms without addressing the neurodegenerative process itself. Therefore, regenerative treatments that provide neurorestorative benefits or neuroprotective effects for the remaining dopaminergic neurons are of increasing interest.

The use of neurotrophic factors is a promising method to arrest the neurodegenerative process. Neurotrophic factors are proteins that promote the development, maintenance, and survival of neurons in the brain. The well-studied glial cell–derived neurotrophic factor (GDNF) has received special attention in several studies due to its specific protective function for dopaminergic neurons in rodent and primate models [8–10]. GDNF has a strong affinity to the GDNF family receptor α1, which is, among other cell types, located on dopaminergic neurons. Activation of this receptor can lead to activation of several pathways with pro-survival effects, such as protecting neurons from apoptotic stimuli like oxidative stress. These effects have been reported in numerous in vivo animal studies [11–13]. A common animal model for PD is the 6-hydroxydopamine (6-OHDA) hemiparkinson lesion model for adult rats. In this model, neurotoxic 6-OHDA is injected locally in one hemisphere (afterwards, called ipsilateral hemisphere), whereas the other hemisphere (contralateral) serves as an internal control [14, 15]. By virtue of 6-OHDA’s high affinity for dopamine transporters, its application leads to a progressive degeneration of dopaminergic neurons in the *substantia nigra* (SN) [16]. The severity of this 6-OHDA disease model can be adjusted by the different anatomical target areas (*striatum*, *medial forebrain bundle*, SN) of the 6-OHDA injection and its concentration. Mild 6-OHDA lesions in the striatum with low 6-OHDA concentrations are ideal to test putative neuroprotective agents since severe lesions show instant and static neuronal loss with no chance for regenerative treatment effects [17]. Intracerebral administration of GDNF showed neuroprotective and neurorestorative effects when given before, concomitantly with, or after 6-OHDA lesion [18–20], resulting in the alleviation of motor dysfunction caused by 6-OHDA [21, 22]. On the basis of these in vivo studies, clinical trials of intracerebral infusion of GDNF protein in PD patients were initiated, though with mixed outcomes [23–25]. A major limiting factor and reason for the heterogeneous outcomes was the insufficient delivery of GDNF into the brain, since GDNF cannot cross the blood–brain barrier [26, 27]. Thus, GDNF must be infused directly into the brain parenchyma, for instance by using minipumps. However, multiple infusion timepoints or high GDNF concentrations are necessary in this approach owing to GDNF’s fast degradation rate [28]. To reduce the resulting dose dumping and side effects of high concentrations, but still deliver sufficient amount of GDNF locally, cells can be used as a continuous GDNF-producing system and injected directly into the desired areas in the brain parenchyma [29, 30].

In particular, mesenchymal stromal cells (MSCs) have emerged as an appropriate option for the treatment of PD. Besides being self-renewal, their neuroprotective and neurorestorative potential as well as their low immunogenicity are beneficial. Some studies reported that differentiation of human MSCs into neurons or neural lineages leads to disease-related improvement, and other, however, suggest that this principle recovery could also be due to the natural secretome of MSCs, which promotes neurogenesis and cell survival [31–33]. To combine the beneficial effects of human adipose tissue–derived MSCs (adMSCs) and GDNF for treating PD, MSCs can be genetically modified to overexpress higher amounts of GDNF. There are several ways to integrate the desired genes of interest into the cells. One promising strategy is to use a DNA transposon–based system such as the Sleeping Beauty (SB) transposon system, consisting of the gene of interest and a transposase gene flanked by two inverted terminal repeats. The transposase is able to recognize these terminal inverted repeats and catalyzes the mobilization of the whole transposon into the cell’s DNA via a “cut-and-paste” transposition mechanism [34]. Typically, two plasmids are co-transfected in the cell: one containing an expression cassette with the gene of interest and the other one encoding the transposase enzyme. Whereas the use of viral vectors like lentiviral vectors for overexpressing GDNF is more common and succeeded in improving PD clinical symptoms of PD rats [35], SB transposons have certain advantages including faster and cheaper production under good manufacturing practices, increased biosafety, and low immunogenicity [36]. Also, since wild-type adeno-associated virus (AAV) infections in the human general population are common, pre-existing circulating AAV-specific humoral components showed significant diminished gene expression and transduction in animal models [63–65]. Therefore, our group has dealt with the transposon and has established itself in this field [51, 52, 61].

Cell survival after transplantation of genetically modified cells may be poor as the damaged area activates the neuroimmune system with its relevant cells like microglia and astrocytes to support healing after transplantation surgery [37, 38]. Biomaterials such as hydrogels can minimize access of inflammatory cells or impede a potential MSC migration before they build their own stable MSC matrix [39, 40]. Additionally, hydrogels provide a scaffold for cell adhesion and growth of transplanted cells without preventing the transport of important molecules like neurotrophic factors or nutrients [41, 42]. One attractive hydrogel candidate for entrapment is the fibrin hydrogel. Its favorable mechanical properties, biocompatibility, biodegradability, and injectability make it a perfect match for entrapping and administrating GDNF-adMSCs. The adaptability of the fibrin hydrogel components and its superior adherence to brain tissue is especially useful in our application [43, 44]. Since it is already commercially available and tested for safety in clinical settings, it can accelerate the potential clinical transition of the proposed therapy concept [45].

In this study, our primary aim was to evaluate if we could successfully transplant GDNF-overexpressing adMSCs with consequential improvement of PD-related pathology in a 6-OHDA rat model. The secondary aim was to identify the potential superiority of GDNF-adMSCs entrapped in fibrin hydrogel compared to GDNF-adMSCs without hydrogel.

## Materials and methods

### In vitro

#### Isolation and cultivation of adMSCs

During tumescent liposuction, primary adMSCs were isolated from human adipose tissue. The procedure was approved by the ethics committee of the Charité – Universitätsmedizin Berlin (approval number EA2/127/07) and by the patient who gave informed consent.

In brief, pre-separation of the harvested adipose tissue aspirate took place overnight at room temperature. The adipose tissue phase was separated from the other phases and centrifuged for 5 min at 350 g. The obtained fatty phase was rinsed and centrifuged three times with equal amounts of phosphate buffered saline (PBS) (Dulbecco’s modified Eagle’s medium, Biochrom AG) and digested with an equal volume of a PBS–collagenase solution, consisting of 0.05% collagenase 1 (Sigma-Aldrich, Ltd.), 200 mM calcium chloride (Merck KGaA), and 0.25% bovine serum albumin (BSA) (BSA FV, Carl Roth GmbH + Co. KG). After incubation for 45 min on a shaker in a humified incubator at 37 °C (5% CO_2_ Heracell) and subsequent two centrifugation washing steps (6 min at 400 g), the adMSC pellet was resuspended and cultivated in standard culture medium (Dulbecco’s modified Eagle’s medium GlutaMAX™ I [Gibco®/Life Technologies], 10% fetal bovine serum [Biochrom AG], and 1% penicillin/streptomycin [10,000 units/ml, Gibco®/Life Technologies]). For cell seeding, a cell suspension aliquot was mixed 1:1 with trypan blue (Gibco®/Life Technologies) and counted with the Countess™ automated cell counter (Invitrogen) or Neubauer counting chamber. Cells were seeded at a density of 2000 cells/cm^2^ and cultivated in the standard culture medium at 37 °C with 5% CO_2_.

For cultivation, standard culture medium was renewed every 3 to 4 days and cells were passaged when reaching 80–90% confluency. To passage adMSCs, the standard culture medium was removed, and cells were washed twice with pre-warmed PBS. Detachment of the cell monolayer was enabled by treatment with 0.05% trypsin/EDTA (Biochrom AG) for 3 min at 37 °C. After detachment, three times the volume of PBS was added to dilute trypsin and the cell suspension was centrifuged (5 min at 300 g). After two washing steps with PBS, adMSCs were reseeded and cultured to a maximum of passage 10.

#### Characterization of adMSCs

AdMSCs at passage 3 were characterized by their ability to differentiate into adipogenic, chondrogenic, and osteogenic lineages. Analysis of relevant surface markers was performed by fluorescence-activated cell sorting (FACS) as described previously [46]. In detail, pellets of 250,000 cells were resuspended in 0.5% BSA/PBS (HyClone™/Cytiva and Merck KGaA) and stained using several mouse anti-human antibodies to a final volume of 100 µl. Antibodies were conjugated either to fluorescein isothiocyanate (FITC) or phycoerythrin (PE). The following antibodies were used to label adMSCs in multiple approaches: CD105 FITC (1:20; TA320325, Acris Antibodies GmbH), CD90 FITC (1:20; 561,969), CD44 FITC (1:20; 555,478), CD73 PE (1:50; 561,014), CD166 PE (1:10; 560,903), CD34 PE (1:50; 550,619), CD14 PE (1:100; 561,707), and CD45 FITC (1:20; 555,482) (all BD Pharmingen™, from BD Biosciences). After incubation on ice for 15 min, cells were centrifuged (10 min, 400 g) at 4 °C and resuspended in 0.5% BSA/PBS to a final volume of 500 µl. Apoptotic cells were excluded from the analysis using additional 0.1 mg propidium iodide. Analysis was performed using FACSCanto™ (BD Biosciences) and the FlowJo™ v10.6.2 software (BD Life Sciences, RRID:SCR_008520).

Multilineage differentiation assays were performed as described previously [46]. Briefly, for all differentiation lineages, adMSCs were cultured in a mix of standard culture medium, 20 mM HEPES (391,340, Merck KGaA), and the corresponding differentiation inducing supplements for a set time period. For adipogenic differentiation, cells were cultured for 15 days using the following adipogenic supplements: 0.1 µM dexamethasone, 0.2 mM indomethacin, 0.5 mM IBMX (all from Sigma-Aldrich, Ltd.), and 2.84 µl/ml insulin actraphane (Novo Nordisk). Adipocytes were identified by Oil Red O staining (O0625, Sigma-Aldrich). For triggering chondrogenesis, adMSCs were cultivated for 28 days in serum-free standard culture medium and a chondrogenic supplement mix containing a universal culture supplement (ITS + Premix Universal), 0.1 µM dexamethasone, 0.17 mM l-ascorbic acid 2-phosphate, 1 mM sodium pyruvate, 0.35 mM l-proline (all Sigma-Aldrich, Ltd.), and 10 ng/ml of the human transforming growth factor‐β3 (100-36E, PeproTech). Chondrocytes were stained with 0.7% safranin O (S8884, Sigma-Aldrich) and 1% alcian blue 8GS (3082.1, Roth). Osteogenic differentiation was induced with osteogenic supplement mix containing 0.1 µM dexamethasone, 0.05 mM l-ascorbic acid 2-phosphate, and 10 mM β-glycerophosphate (all from Sigma-Aldrich, Ltd.) and cultured for 28 days. Osteocytes were stained with nitroblue tetrazolium/5-bromo-4-chloro-3-indolyl phosphate (11,697,471,001; Roche), the von Kossa staining method, and 0.5% alizarin red S (A5533, Sigma-Aldrich, Ltd.).

#### Live/dead staining with propidium iodide/fluorescein diacetate

To determine viability, hMSCs encapsulated in fibrin hydrogel were stained with propidium iodide/fluorescein diacetate (PI/FDA). Stains were done on 2 drops per variant of encapsulation, on days 1, 2, 3, 7, 10, and 14 after encapsulation, using different drops on each sampling day. The medium was removed, and each well was rinsed once with PBS. Each well was covered with 300 µl of an FDA solution (3 µg/ml in PBS) and incubated at 37 °C. The solution was removed, and each well was rinsed again with PBS. Three hundred microliters of PI solution (0.1 mg/ml) was added. After 2 min, the PI solution was removed and each well was rinsed threefold with PBS. The plate was then documented via a fluorescence microscope (excitation of FDA is done at 460–490 nm, while emission cutoff is at > 520 nm). Excitation of PI is done at 480–550 nm, and emission cutoff is at > 590 nm.

#### Vector design

AdMSCs were transfected using two plasmid vectors: the SB transposase vector and the SB transposon vector. The gene of interest, either isoform 1 of human GDNF or enhanced green fluorescent protein (eGFP), was encoded in the transposon vector, regulated by the core promoter for human elongation factor 1α. It was also regulated by a puromycin selection cassette, controlled by the simian virus 40 enhancer. EGFP was used as the reporter gene for testing transfection procedure and transposon compatibility. The pCDNA3.1 plasmid (RRID:Addgene_79663) was used as an insert for the design of the SB transposon vector. The transposase vector pCMV(CAT)T7-SB100, expressing a hyperactive SB100X transposase, was used for the integration of the transposon vector. The pCMV(CAT)T7-SB100 was gifted by Zsuzsanna Izsvak (Addgene plasmid #34879; http://n2t.net/addgene:34879; RRID:Addgene_34879).

#### Nucleofection and selection of adMSCs

AdMSCs were transfected via nucleofection using the Amaxa® Nucleofector™ 2b Device according to the protocol of the Human MSC Nucleofector® Kit (both from Lonza Group AG). The SB transposon vector was mixed with the SB transposase vector in a ratio of 9:1, and 4 µg of this plasmid mixture was transferred with 600,000 cells/100 µl into the kit’s electroporation cuvette and electroporated using program C-17. Afterwards, cells were diluted in 500 µl pre-warmed standard culture medium and further incubated at 37 °C with 5% CO_2_. Transfected cells were selected 3 days after transfection by adding 1.5 µg/ml puromycin (InvivoGen) to the culture medium for seven consecutive days. The selection was maintained by replacing the selection medium every 3 to 4 days. No exact cell counting was done during the antibiotic selection and until reaching cell confluency, which was reached around 3 weeks after transfection (see Fig. 2A). After selection, transfected adMSCs were further cultured as aforementioned. Throughout the subsequent cell cultivation, viability was measured weekly and was in a range of 88–97% viable cells and cells showed vitality in reaching continuously cell confluence 1 week after passaging.

In vitro, eGFP expression levels were monitored at 10 days and 2 weeks, 3 weeks, 4 weeks, and up to 8 weeks post-transfection and captured at × 10 magnification using an Eclipse Ti-U microscope connected to a DS-Qi2 camera and the software NIS-Elements BR 4.51 (all from Nikon, NIS-Elements Basic Research, RRID:SCR_002776).

#### Enzyme-linked immunosorbent assay

To test in vitro expression of GDNF prior to in vivo transplantation, samples of cell culture medium from GDNF-adMSCs and naïve adMSCs were collected at three different timepoints (1 h, 24 h, and 48 h after replacing medium) before passaging and stored at − 80 °C until further analysis. GDNF concentrations of samples were measured using a commercially available human GDNF enzyme-linked immunosorbent assay (ELISA) kit (EHGDNF, Thermo Fisher Scientific GmbH) according to the manufacturer’s protocol. The obtained GDNF amounts (pg/ml) were further calculated with cell numbers of the respective timepoints. Results are therefore given in ng/120,000 cells, which is the number of transplanted cells per animal.

### In vivo

#### Animals

This study was approved by the local authority LAGeSo (Landesamt für Gesundheit und Soziales, Berlin; approval numbers G0385/17 and G0132/19). All animal procedures were performed in accordance with the European Communities Council Directive of 22 September 2010 (2010/63/EU). The study was carried out, and the manuscript was written in compliance with the ARRIVE guidelines by the National Centre for the Replacement, Refinement and Reduction of Animals in Research (NC3Rs).

In total, 88 adult male Wistar Han rats (purchased at 6–7 weeks of age, weighing 220–240 g, Charles River Laboratories, RGD Cat# 2,308,816; RRID:RGD_2308816) were randomly assigned to cages with the help of an online random number generator (GraphPad: https://www.graphpad.com/quickcalcs/randomize1/). They were group housed in standard type IV cages in a temperature (20–24 °C), humidity (relative humidity 45–65%), and light (12-h light/dark cycle)–controlled environment in a specific pathogen-free facility. Animals had ad libitum access to rodent laboratory chow and autoclaved tap water and were acclimatized for 1 week prior to the beginning of the experiments.

#### Study design

**Fig. 1:**
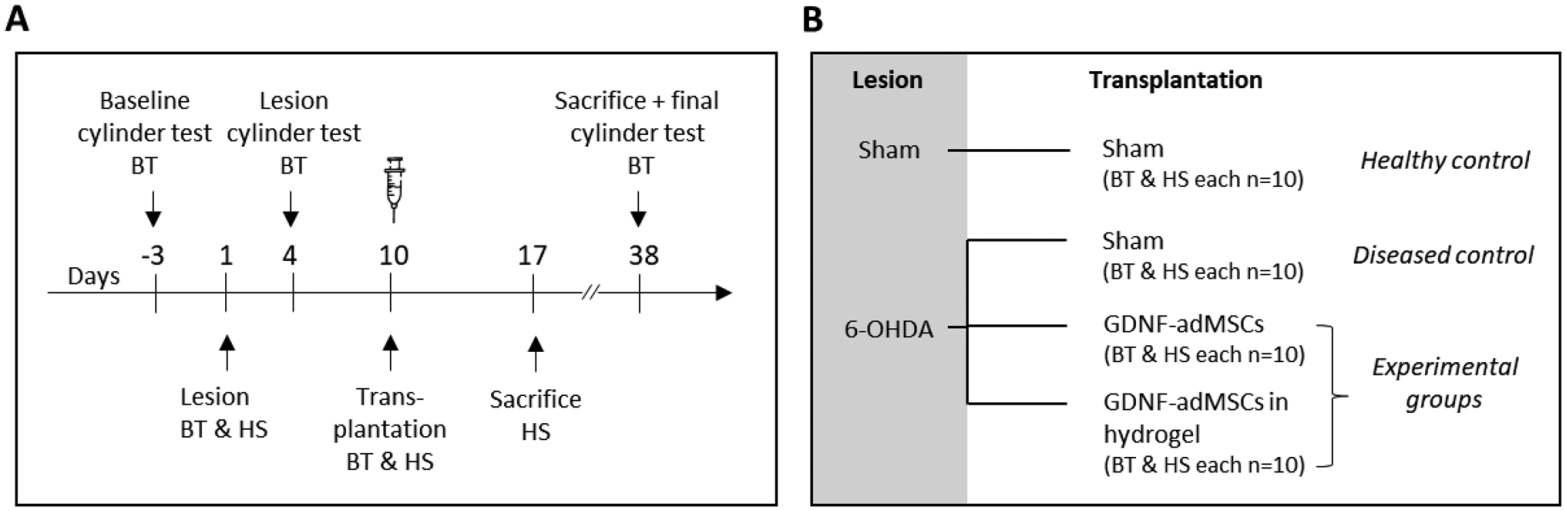
Study design. **A**, **B** Animals were assigned to four groups; for each group, 10 animals underwent behavioral testing (BT) and were sacrificed 1 month after transplantation surgery, and the other 10 animals were sacrificed 1 week after transplantation surgery and were used for establishment of histologic stainings (HS). All animals received a lesion surgery with either saline (healthy control) or 6-hydroxydopamine (6-OHDA) (all other groups) and a transplantation surgery with saline (healthy and diseased control), bare GDNF-adMSCs, or GDNF-adMSCs entrapped in hydrogel (experimental groups, treatment marked with a syringe). BT animals underwent cylinder tests 3 days before lesion (baseline), 4 days after lesion (lesion), and 1 month after transplantation surgery (final). Sham, saline treatment

Animals were assigned to four different groups with 20 animals per group: healthy and diseased control groups, where either saline (sham) or 6-OHDA was given for lesion surgery and only saline was applied during transplantation surgery. Experimental groups consisted of animals that all were lesioned with 6-OHDA and received GDNF-adMSC transplantation either with or without hydrogel (Fig. 1B). In each group, 10 animals were used for establishing histological staining (HS) without behavioral testing and 10 animals underwent behavioral testing (BT). Before starting the first surgeries, BT animals underwent a baseline control cylinder test 3 days before 6-OHDA lesion. On lesion surgery day, BT and HS animals received a single dose of either 6-OHDA to induce hemiparkinsonism (diseased controls) or saline (healthy controls) into the left striatum. Three days post-lesion surgery, the lesion effect of BT animals was pre-checked via lesion cylinder test. Nine days after lesion surgery, transplantation surgery was conducted using a striatal injection of either saline, GDNF-adMSCs, or GDNF-adMSCs entrapped in hydrogel into the same location in BT and HS animals. HS animals were sacrificed 1 week after transplantation surgery, whereas BT animals had their final cylinder test 1 month after transplantation before they were sacrificed and stained for immunohistological analyses. All immunohistological and behavioral analyses were performed by an experimenter blinded to the group allocation, and data was also analyzed blinded. Due to the handling differences (treatment with and without hydrogel), the experimenter could not be blinded to the group allocation during surgeries.

#### Stereotaxic surgery

Rats from all groups received anesthesia, induced with oxygen-containing 5% isoflurane (CP-Pharma Handelsgesellschaft mbH), and maintained with oxygen containing 2–3.5% isoflurane as needed. Oxygen flow was set to 1.5 l/min, and a rat gas anesthesia mask was used. After subcutaneous injection of 2 mg/kg meloxicam (Metacam®, Boehringer Ingelheim), an eye ointment (Dr. Winzer Pharma GmbH, Pan-Ophtal Gel) containing 5% dexpanthenol was applied and cessation of reflexes was ensured. Animals were secured in a stereotaxic frame (TSE Systems), respiration was monitored, and hypothermia was prevented by using a heating pad (TRIXIE Heimtierbedarf GmbH & Co. KG). The animal’s head was first shaved, disinfected with ethanol, and cranium exposed (skin incision of 1 cm). For cranial trepanation, a mini drill (HOBBY DRILL Typ 2, Donau Elektronik GmbH) was used. In lesion surgeries, the location of the injection was at anteroposterior + 1 mm, mediolateral + 3 mm, and dorsoventral − 5 mm from bregma [47]. For generating the unilateral mild retrograde lesion, a single dose of 12 µg 6-OHDA (Sigma-Aldrich, Ltd.), dissolved in 2 µl 0.9% saline and 0.1% ascorbic acid (Sigma-Aldrich, Ltd.), was infused into the left striatum through a 26-gauge syringe (Hamilton Company). After applying the neurotoxin, the syringe was kept in place for additional 5 min before being retracted. Healthy control animals received 2 µl saline.

For transplantation, a 22-gauge syringe (Hamilton Company) was used since a bigger diameter was needed to not damage the adMSCs during the injection [48]. Here, the location of the injection anteroposterior and mediolateral was the same as for the lesion surgery, the dorsoventral coordinates differed. Firstly, the needle was inserted at − 5.8 mm dorsoventral to create a reservoir for the different transplants. Directly before injection, the syringe was set to + 4.3 mm dorsoventral for transplantation of 5 µl of 0.9% saline (healthy and diseased control) or 5 µl of GDNF-adMSCs was harvested and resuspended either in 0.9% saline or in a hydrogel. To entrap GDNF-adMSC, cells were suspended in aliquots of the commercially available Tisseel Fibrin Sealant (TISSEEL, Baxter). The fibrin sealant kit consists of 2 components: the sealer protein solution, containing 91 mg/ml human fibrinogen, and the thrombin solution, containing 500 units/ml human thrombin. Both components are ready-to-use frozen solutions, each pre-filled into one side of a dual-chambered syringe. Since the dual-chambered syringe is designed for human application so that both components get mixed in a joining piece before being dripped through the cannula, the provided syringe was not used. Each component from the dual-chambered syringe was filled into separate reaction tubes, where aliquots were taken. To avoid premature polymerization of the fibrin hydrogel in the injection cannula, the fibrinogen and thrombin components were administered via a 3-layered system. Similar to Moloney et al. (2015), 2 µl of fibrinogen component was physically separated from the 1 µl thrombin component with 1 µl PBS layer in between. In the adMSC + hydrogel group, 120,000 GDNF-adMSCs were resuspended in the fibrinogen phase whereas the same number of cells was suspended in 0.9% saline for the GDNF-adMSC group without hydrogel [45]. After injection of transplants, the syringe remained in position for 10 min to enable enough time for hydrogel polymerization, before being retracted. The skin of the head was sutured with surgical needle-equipped sutures (Ethicon Ethilon Polyamide 6 3–0, Johnson & Johnson GmbH), and 2 mg/kg meloxicam was applied on three consecutive days after surgery.

#### Cylinder test

The cylinder tests were performed according to Schallert et al. (2000) to assess locomotor activity at different timepoints [49]: the baseline test was performed at 3 days prior to lesion surgery, lesion tests were performed at 3 days after lesion surgery, and the final tests were performed 1 month after transplantation surgeries. Cylinder behavioral tests were conducted in a transparent glass cylinder (diameter: 25 cm, height: 30 cm) in the animal facility during dark phase under low-light conditions, ensuring adequate locomotor activity. Animals were placed in the glass cylinder, and right forepaw touches (referred to as contralateral paw, controlled by lesioned left hemisphere) for a minimum of 20 touches or a maximum of 10 min were recorded using a Legria HF R506 camera (Canon). Recorded videos were analyzed manually using Kinovea Player 0.8.15 (Joan Charmant & Contrib.) [50]. The examining person was blinded to group allocation. Data are presented as the ratio of wall contacts with the contralateral paw to the ipsilateral paw (internal control).

#### Magnetic resonance imaging data acquisition

To investigate fibrin hydrogel stability, additional eight animals were anesthetized with oxygen containing 2–3.5% isoflurane and placed in a 7-T small animal magnetic resonance imaging (MRI) (Bruker, BioSpec 70/20 USR, Ettlingen, Germany), running with ParaVision 6.1 software (Bruker Corporation, RRID:SCR_001964). Each subject was placed on an animal carrier which provided fixation via tooth bar and ear holders to minimize movements while measuring. Respiratory rate was monitored throughout the procedure, and animals were placed on a bed with circulating heated water to maintain constant body temperature during the scan. A T2-weighted image set was acquired with 52 contiguous slices with a slice thickness of 0.5 µm. Two animals with fibrin hydrogel transplant had MRI on the day of transplantation surgery, two animals had MRI 1 week after transplantation surgery, and two animals had MRI 1 month after transplantation surgery. Two animals without hydrogel but PBS as control had MRI on the day of transplantation surgery. These animals did not undergo any behavioral tests or other testing and were only used for MRI.

#### Perfusion and tissue processing

Animals received terminal anesthesia with oxygen-containing 5% isoflurane and were injected with 10 mg/kg xylazine hydrochloride (Xylavet®, CP-Pharma Handelsgesellschaft mbH) and 80 mg/kg ketamine hydrochloride (Ketamin Inresa, Inresa Arzneimittel GmbH). After respiratory arrest and cessation of reflexes were observed, animals were transcardially perfused with 250 ml of ice-cold 0.1 M PBS followed by 250 ml of 4% paraformaldehyde dissolved in 0.1 M PBS. Whole brains were rapidly removed, post-fixed in 4% paraformaldehyde for 2 h, and dehydrated in 30% aqueous sucrose solution. After 48 h of dehydration at 4 °C, brains were frozen in 2-methylbutane (Sigma-Aldrich, Ltd.) at − 68 °C for 50 s. Brains were cut into 40-µm coronal cryosections using a freezing stage microtome (Leica CM1850 UV, Leica Biosystems). In doing so, the contralateral (non-operated) hemisphere was labeled by longitudinal punctuation with a 26-gauge cannula. Sections were stored in cryoprotectant solution at 4 °C (25% v/v glycerol, 25% v/v ethylene glycol, and 50% v/v 0.1 M PBS).

### Ex vivo

#### Immunohistochemistry

Free-floating immunohistochemistry against human GDNF was performed with sections of a one-in-twelve series containing the striatal *caudoputamen* (CPu). Tyrosine hydroxylase (TH) was stained in a one-in-six series of CPu and SN brain sections. Stainings were performed as previously described [51, 52]. In brief, endogenous peroxidase activity was quenched using a solution of 0.6% H_2_O_2_ (Carl Roth GmbH + Co. KG) in 0.1 M PBS for 30 min. Unspecific antibody binding was blocked using 3% donkey serum and 0.1% Triton X-100 (both Sigma-Aldrich, Ltd.), diluted in PBS (from now on called PBS +) for 30 min. Sections were incubated overnight at 4 °C with the following primary antibodies in PBS + : mouse anti-TH (1:10,000, T1299, Sigma-Aldrich, Ltd.) or goat anti-GDNF (1:200, BAF212, R&D Systems). Subsequently, sections were washed with PBS and re-blocked with PBS + for 25 min, priming for secondary antibody incubation. Appropriate biotinylated secondary antibodies were added to the sections for 2 h at room temperature: donkey anti-mouse-biotin-SP (1:250, 715–065-151) or donkey anti-goat-biotin-SP (1:250, 705–065-147, all from Dianova GmbH). A streptavidin–biotin–horseradish peroxidase solution (Vectastain, ABC Elite Kit, Vector Laboratories) was added to the sections and incubated for 1 h. For visualization of staining, 3,3′-diaminobenzidine tetrahydrochloride hydrate and 8% nickel chloride were used (both from Sigma-Aldrich, Ltd.). After staining, sections were mounted on slides, dehydrated in an ascending ethanol series, and coverslipped (ProTaqs® Clear, Quartett GmbH).

To visualize different transplant components, immunofluorescent staining against different markers was performed. The nuclear mitotic apparatus protein 1 (NuMa) antibody was used as a marker for the human origin of the adMSCs, the fibrin antibody for visualizing the hydrogel. Staining of these markers and of human GDNF was performed according to an established protocol [52, 53]. Briefly, striatal sections were blocked with PBS + for 30 min and incubated overnight with the primary antibodies: rabbit a-nuclear mitotic apparatus protein antibody (1:100, ab86129, Abcam), mouse a-fibrin (1:100, orb302441, Biorbyt), and goat a-GDNF (1:200, BAF212, R&D Systems) at 4 °C. The next day, re-blocking with PBS + for 25 min was followed by light-protected incubation with Alexa Fluor 555 (donkey anti-rabbit, 1:20, SBA-6442–32, Dianova), Alexa Fluor 350 (donkey anti-mouse, 1:100, Thermo Fisher Scientific GmbH), and Alexa Fluor 488 (donkey anti-goat, 1:500, Thermo Fisher Scientific GmbH) for 4 h and 4′,6-diamidino-2-phenylindole (DAPI) (1:1000, D1306, Thermo Fisher GmbH Scientific) for 10 min. Finally, brain sections were mounted on microscope slides and coverslipped (Aqua-Poly/Mount, Polysciences Europe GmbH).

#### Immunohistological analyses

To assess the amount of TH-positive (TH +) cells in six nigral serial sections per animal, the stereological optical fractionator method was used [54, 55]. Cells were stereologically extrapolated using the “Optical Fractionator Probe” of Stereo Investigator 10.40 software (MicroBrightField, Stereo Investigator, RRID:SCR_018948) with a grid size of 200 × 200 µm and a counting frame of 100 × 100 µm. The analysis was performed using a × 40 objective of a BX53 microscope (Olympus) attached to a DV‐47d camera (MicroBrightField). Results are expressed as the ratio of contralateral to ipsilateral cell numbers.

Striatal and nigral photos were captured using a light microscope (Zeiss Axio, Carl Zeiss AG) connected to ProgRes® CapturePro 2.8.8 (Jenoptik AG). Images were converted using ImageJ 1.52a (Wayne Rasband, ImageJ, RRID: SCR_003070).

Fluorescent transplant photos were visualized with an Olympus CKX41 microscope combined with a reflected fluorescence microscopy system (Olympus Corporation). Photos were captured using a ProgRes® CapturePro 2.8.8 camera combined with the ProgRes CapturePro 2.10 software (both Jenoptik AG), and overlays were merged by the use of ImageJ 1.52a (Wayne Rasband, ImageJ, RRID: SCR_003070).

#### Measurement of inflammatory cytokine levels of striatal transplantation slices

To analyze inflammatory reactions at the ipsilateral transplantation site for each rat, full-length proteins of two formalin-fixed striatal slices displaying the transplantation site were lysed with the help of a homogenizer (Witeg, HG-15A). In total, proteins of 5 animals per group were isolated using the PreAnalytiX Supplementary Protocol: “Purification of full-length proteins from OAXgene Tissue fixed and stabilized (PF) tissue samples” of the Qproteome FFPE Tissue Kit (Qiagen GmbH). In brief, samples were homogenized in extraction buffer EXB Plus, heated to 95 °C for 10 min, and incubated on ice for 5 min. After cooling down, samples were centrifuged (5 min, 14,000 g, at 4 °C) and the supernatant was collected. The Bradford method (Coomassie Bradford Protein Assay Kit, Thermo Fisher Scientific GmbH) was used to measure total protein concentration, samples were diluted 1:15 with Aqua Dest, and a final volume of 10 µl diluted protein extract was analyzed in triplicates as described in the kit’s manual.

Cytokine levels of protein samples were analyzed in duplicates with the V-PLEX Proinflammatory Panel 2 Rat Kit (Meso Scale Diagnostics LLC) according to the manufacturer’s manual. Concentration levels of interferon-γ (IFN-γ), keratinocyte chemoattractant/human growth-regulated oncogene (KC/GRO), tumor necrosis factor-α (TNF-α), and interleukins (ILs) IL-1β, IL-4, IL-5, IL-6, IL-10, and IL-13 were measured and calculated with a 4-parameter logistic model with 1/*y* square weighting except for KC/GRO, since concentration happened to be beneath the kit’s detection limit. The amount of cytokines in pg was normalized to the total amount of proteins in mg.

#### Statistical analyses

All data sets were analyzed with SPSS Statistics 25 (IBM, SPSS Statistics, RRID:SCR_019096) for parametric testing. Grubb’s test was used for outlier testing before each statistical test and resulted in only 2 outliers in the analysis of cylinder tests (Fig. 7) in the GDNF-adMSC group and the diseased control group. Normal distribution was investigated by D'Agostino–Pearson omnibus *K*^2^ test, and equality of variances was tested with the Brown–Forsythe test. If the criteria for parametric testing were not met, nonparametric tests were chosen for further analysis. Data analysis were conducted with RStudio 1.2.5033 (RStudio, RRID: SCR_000432) using the packages nparcomp or nparLD [56, 57]. The mctp function of the nparcomp package was used to perform nonparametric multiple contrast test (type Tukey–Kramer) between groups, whereas the nparLD package was used to perform nonparametric ANOVA-type statistics of data sets that include repeated measures with multiplicity-adjusted *p* values. Results were plotted with GraphPad Prism 8.4.2 (GraphPad Prism, RRID: SCR_002798), usually using boxplots with interquartile ranges (IQR) from the 25^th^ to 75^th^ percentile with whiskers (min to max) and median. *p* values less than 0.05 were considered statistically significant.

## Results

### Verification of MSC phenotype and differentiation potential

Generic stem cell surface antigens of donor cells were analyzed by flow cytometry. Human adMSCs were positive for the presence of common MSC markers, CD105, CD90, CD44, CD73, and CD166 (Supplementary Fig. S1A). Cells were negative for the hematopoietic antigen CD34 as well as leukocyte antigens such as CD45 and CD14. To verify the multipotent differentiation potential of the adMSCs, the cells were cultured in differentiation media with different supplements and stained for specific characterization details like lipid droplets (adipocytes), calcium-rich mineral deposition (osteocytes), and proteoglycan deposition (chondrocytes) (Supplementary Fig. S1B). Cultivation in adipogenic differentiation medium led to multiple lipid vacuoles stained by Oil Red O staining as a marker for adipogenic MSC differentiation. Osteogenic lineage was confirmed by Alizarin and Von Kossa staining, showing the presence of alkaline phosphatase and bone mineralization processes. Chondrogenesis was inducible with chondrogenic supplements in dense adMSC pellet cultures with proteoglycan deposition stained by alcian blue 8GS and collagen type II deposition detected with collagen type II antibodies. Neither adipogenic, osteogenic, nor chondrogenic differentiation of adMSCs was induced in adMSC controls lacking respective differentiation media. Overall, adMSCs showed typical surface antigen pattern and multilineage differentiation potential.

### Viability of adMSCs entrapped in fibrin hydrogel

In in vitro preliminary tests, we examined the distribution and viability of entrapped adMSCs in fibrin hydrogel (Supplementary Fig. S6 and Fig. S7). The drops were generated with a syringe having the same inner diameter as the syringes which were used for transplantation surgeries. The drops contained adMSCs with high viability and also predominantly homogenous distribution. For comparison, in Fig. S6B, the fibrin hydrogel was diluted with hyaluronic acid and showed insufficient polymerization and a different distribution pattern. In Fig. S7, syringe was incubated 10 min with adMSC–fibrin hydrogel, reconstructing the case that the fibrin hydrogel was already polymerized in the syringe. This created an elongated hydrogel loop with initial lower viability, probably caused by higher mechanical stress within the firmer hydrogel during pushing through the syringe. However, after three additional days, viability levels recovered to approximated 95%.

### Stable overexpression of nucleotransfected adMSCs

**Fig. 2:**
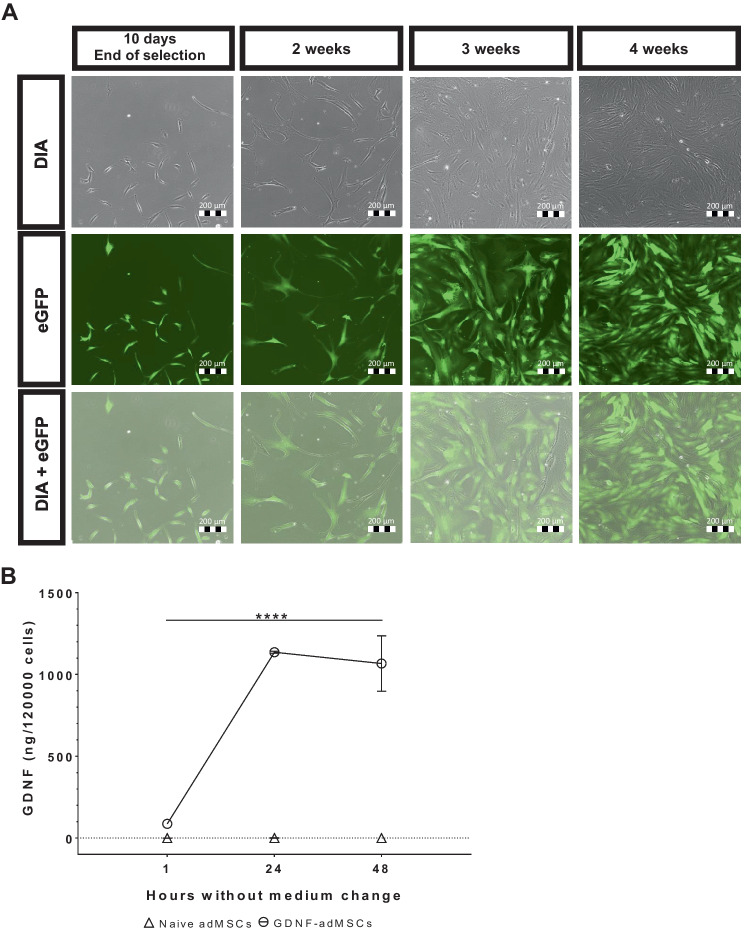
In vitro analysis of GFP-adMSCs and GDNF-adMSCs. **A** Observing Sleeping Beauty (SB) transposon stability and functionality of eGFP-adMSCs via fluorescence microscopy. Puromycin-selected enhanced green fluorescent protein (eGFP)-positive adipose tissue–derived mesenchymal stromal cells (adMSCs) were visible up to 4 weeks after transfection. **B** Evaluation of glial cell line–derived neurotrophic factor (GDNF) secretion was done by measuring GDNF concentration in GDNF-adMSC cell culture medium. GDNF-adMSCs produced significantly more GDNF compared to naive adMSCs at all timepoints. Dotted line indicates the detection limit of the assay (0.00274 ng/ml). Due to the transplantation of 120,000 GDNF-adMSCs per transplantation surgery, data is given in ng/120,000 cells as mean ± SD. Results from the nonparametric multiple comparison are depicted with asterisks: ****p < 0.0001. DIA, transmitted light

An optimized transfection procedure and transposon compatibility were established using enhanced green fluorescent protein as a reporter gene (eGFP-adMSCs). Expression was monitored at four timepoints: 10 days, 2 weeks, 3 weeks, and 4 weeks after transfection (Fig. 2A). Due to antibiotic selection, the cell confluency was low at 10 days after transfection. However, all remaining eGFP-adMSCs were fluorescent at this time point and stayed fluorescent at all other timepoints. Cells showed visible eGFP fluorescence in the time-relevant phase for in vivo studies (1 month) and even up to 8 weeks after transfection (Supplementary Fig. S2A). After establishing an efficient transfection procedure, GDNF was used as the gene of interest for generation of GDNF-adMSCs. GDNF secretion from GDNF-adMSCs was measured in cell culture medium using ELISA (Fig. 2B). ELISA results showed a time-dependent significant increase in GDNF levels in a medium of GDNF-adMSCs, which was higher than GDNF secretion from control-naïve adMSCs at all timepoints (*F* (1.00, 2.00) = 72.00, ****p < 0.0001). Naïve adMSCs secreted GDNF at levels below the detection limit of the ELISA (0.00274 ng/ml) at all timepoints, whereas the GDNF concentration of GDNF-adMSC medium increased from 87.2 ± 5.2 ng/120,000 cells 1 h after transfection to 1066.8 ± 169.4 ng/120,000 cells after 48 h, proving successful GDNF synthesis by GDNF-adMSCs.

### Transplantation of GDNF-adMSCs in vivo and detection of GDNF-adMSCs in the experimental groups with hydrogel

**Fig. 3:**
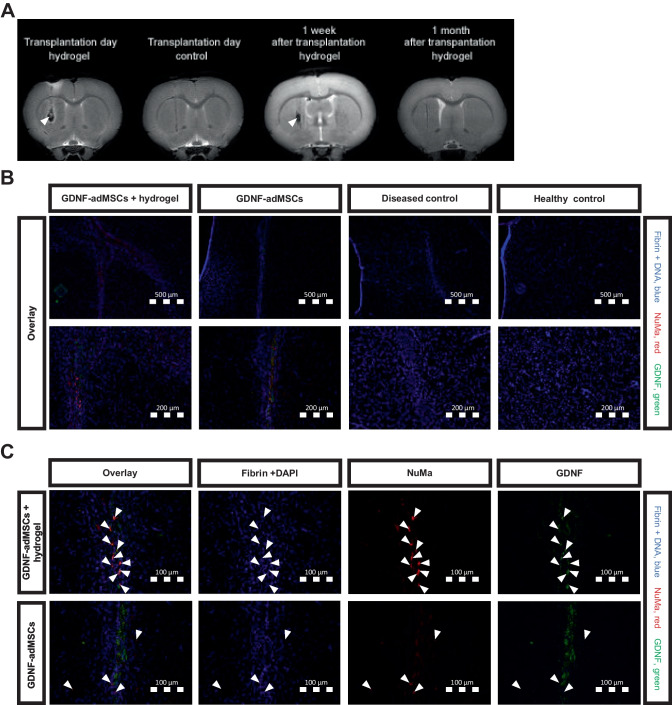
Visualization of transplant components in vivo. **A** Fibrin hydrogel appearance in magnetic resonance imaging (MRI) T2 scan at different timepoints after transplantation surgery. White arrowheads point to potential hydrogel remnants or hydrogel cavities. **B** Detection of GDNF-adMSCs (nuclear mitotic apparatus protein 1 (NuMa), red) and secreted glial cell line–derived neurotrophic factor (GDNF) (green) in both experimental groups after 1 month, and 4′,6-diamidino-2-phenylindole (DAPI) (blue) staining for marking DNA of cell nucleus or fibrin (blue). There is no NuMa or GDNF signal in control groups. Two magnifications and only overlays are shown. **C** The highest magnification of both experimental groups with separate channels and overlay. White arrowheads mark stained GDNF-adMSCs

The hydrogel transplantation was pretested to confirm the location, correct position, and stability of the hydrogel in vivo. To detect the hydrogel, an MRI T2 scan was performed in four rats to illustrate high water content of hydrogel as bright signal. Pure hydrogel without cells showed a bright signal on transplantation day and a lower signal 1 week after transplantation (Fig. 3A, white arrowheads). The control group, only injected with PBS, showed no hydrogel signal or cavity signal, but a dark signal in the injection canal. Likewise, 1 month after transplantation with pure hydrogel, there was neither a dark cavity nor a bright hydrogel. Only the injection canal similar to the sham transplantation control was visible, indicating the degradation of the hydrogel.

Although the hydrogel was likely degraded 1 month after transplantation, GDNF-adMSCs could be visualized via immunostaining ex vivo in both experimental groups treated with GDNF-adMSCs at 1 month post-transplantation (Fig. 3B). In both groups, human origin of adMSCs was detectable via staining by NuMa (red fluorescence) and human GDNF (green fluorescence) was detectable inside and outside of these GDNF-adMSCs. In general, more GDNF-adMSCs with a brighter NuMa signal were visible in entrapped GDNF-adMSC groups compared to the group treated with GDNF-adMSCs alone (Fig. 3C, white arrows, and Supplementary Fig. S3). Yet, the amount of NuMa + adMSCs decreased in both experimental groups after 1 month, compared to respective 1-week groups. In the diseased control and healthy control, neither NuMa + nor GDNF + cells were present (Fig. 3B and Supplementary Fig. S3). In all four groups, fibrin hydrogel was not detectable 1 month after transplantation, consistent with the MRI results. As shown in Supplementary Fig. S3, the hydrogel was detectable in animals with entrapped GDNF-adMSC transplants 1 week after transplantation (blue fluorescence, Supplementary Fig. S3). In summary, GDNF-adMSCs with or without hydrogel were successfully transplanted in vivo, GDNF secretion was evident, and cells and GDNF were still detectable, albeit in reduced levels, 1 month after transplantation surgery.

### Concentrated and prolonged GDNF distribution of entrapped GDNF-adMSCs

**Fig. 4:**
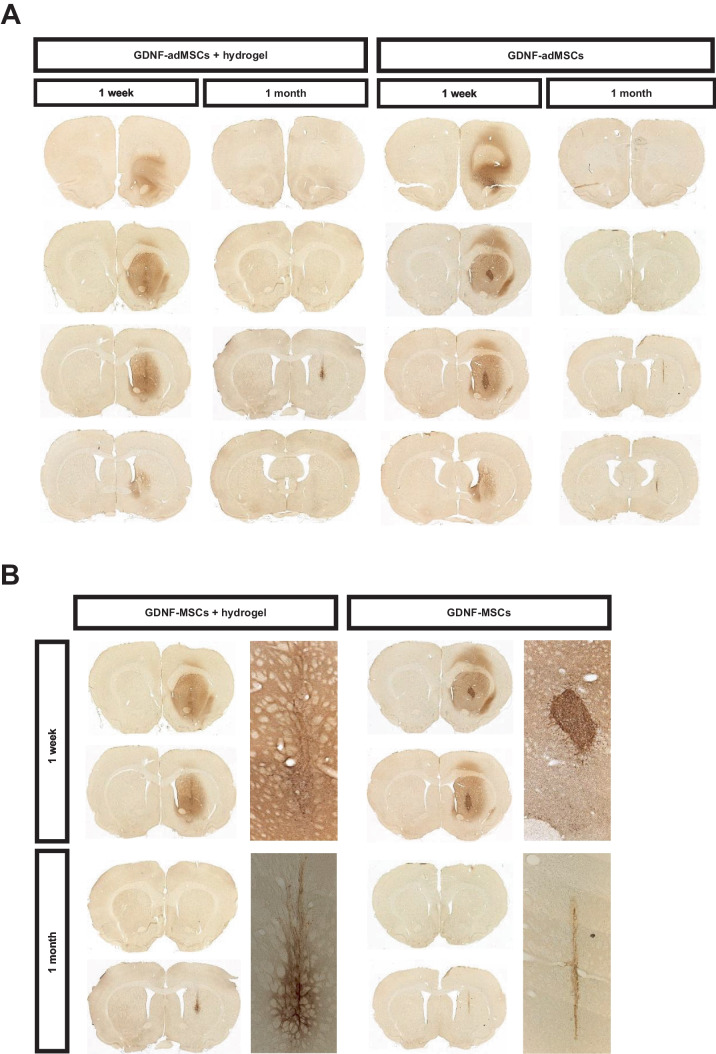
Glial cell line–derived neurotrophic factor (GDNF) distribution ex vivo. DAB-GDNF stainings of sequential striatal slices. **A** Spatial distribution of GDNF 1 week and 1 month after transplantation surgery in the experimental groups. **B** Detailed photomicrographs from the transplantation site. adMSCs, adipose tissue–derived mesenchymal stromal cells; DAB, 3,3′-Diaminobenzidine

Distribution of secreted human GDNF from GDNF-adMSCs was analyzed ex vivo via GDNF immunostaining. While there was no GDNF detectable in diseased and healthy controls (Supplementary Fig. S4), GDNF-adMSCs with and without hydrogel were capable of secreting GDNF into the striatum (Fig. 4A, B). In animals with the sole GDNF-adMSC transplant, GDNF secretion outside the striatum was also observed (Fig. 4A). There was an intensive GDNF signal in the middle of the transplantation region in some animals of the GDNF-adMSC group. However, the presence of GDNF strongly reduced after 1 month. In contrast, the GDNF secretion of the entrapped GDNF-adMSCs was more restricted to the striatum, and GDNF protein was still present after 1 month. Interestingly, in some animals the TH + and GDNF + expression patterns match perfectly, showing functional GDNF and TH expressing transplanted cells (Supplementary Fig. S5).

### Significant reduction of pro-inflammatory IL-1β cytokine levels in entrapped GDNF-adMSC 1 month after transplantation

**Fig. 5:**
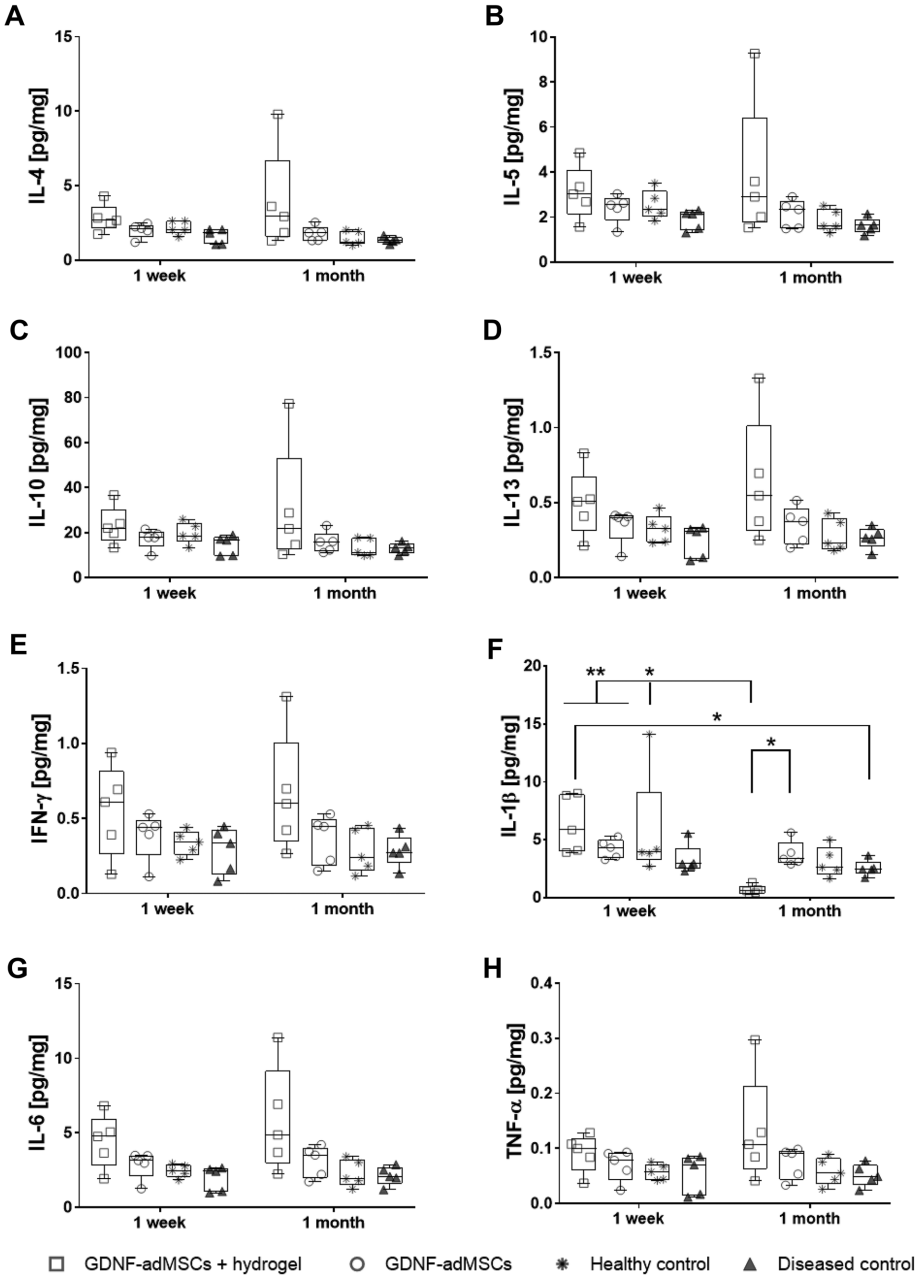
Measurement of inflammatory cytokines levels at transplantation site. Cytokine levels measured by multiplex enzyme-linked immunosorbent assay (ELISA). Anti-inflammatory cytokines (**A**–**D**) tended to have higher levels in the adMSC experimental groups than in the control groups regardless of the timepoint of the analysis. Several pro-inflammatory cytokines (**E**, **G**, **H**) of the adMSC groups showed a trend toward having higher levels than controls, except for IL-1β (**F**). IL-1β levels from the GDNF-adMSC + hydrogel group 1 month after transplantation surgery were significantly lower compared to their controls and to experimental groups 1 week after transplantation surgery. The amount of cytokines in pg was normalized to the total amount of proteins in mg. Boxes indicate 25^th^ and 75^th^ percentiles and median, and whiskers indicate min and max values; *p < 0.05, **p < 0.01. IFN-γ, interferon-γ; IL, interleukins; TNF-α, tumor necrosis factor-α

To evaluate the possible immunomodulatory effects of entrapped or non-entrapped GDNF-adMSCs at the transplantation site, protein extracts from hemisphere slices containing cell transplants were isolated. The main anti-inflammatory cytokines IL-4, IL-5, IL-10, and IL-13 (Fig. 5A–D) showed a trend toward higher levels in the hydrogel-entrapped GDNF-adMSC group at 1 week and 1 month after transplantation surgery compared to the other groups at the same timepoint, but the differences were not significant between groups or over time.

The same trend was observed for the pro-inflammatory cytokines IFN-γ, IL-6, and TNF-α (Fig. 5E, G and H), with IL-1β being the only exception. At 1 month post-transplantation, entrapped GDNF-adMSCs showed the lowest level of IL-1β in all groups (Fig. 5F). Moreover, the entrapped GDNF-adMSC group showed a significant decrease of 89% in IL-1β expression levels compared to the earlier 1 week timepoint (GDNF-adMSCs + hydrogel 1 month vs. GDNF-adMSCs + hydrogel 1 week, **p = 0.003). Similarly, entrapment of GDNF-adMSCs led to significantly lower IL-1β levels compared to the non-entrapped GDNF-adMSCs 1 month after transplantation (decrease by 83%, GDNF-adMSCs + hydrogel 1 month vs. GDNF-adMSCs 1 month, *p = 0.02). In summary, IL-1β levels of GDNF-adMSC + hydrogel group 1 month after transplantation were either on the same level like the other groups or significantly lower, suggesting a potential cumulative immunomodulatory mechanism of the GDNF-adMSCs due to hydrogel entrapment.

### Increased presence of striatal and nigral TH + fibers and cells in GDNF-adMSC groups

**Fig. 6:**
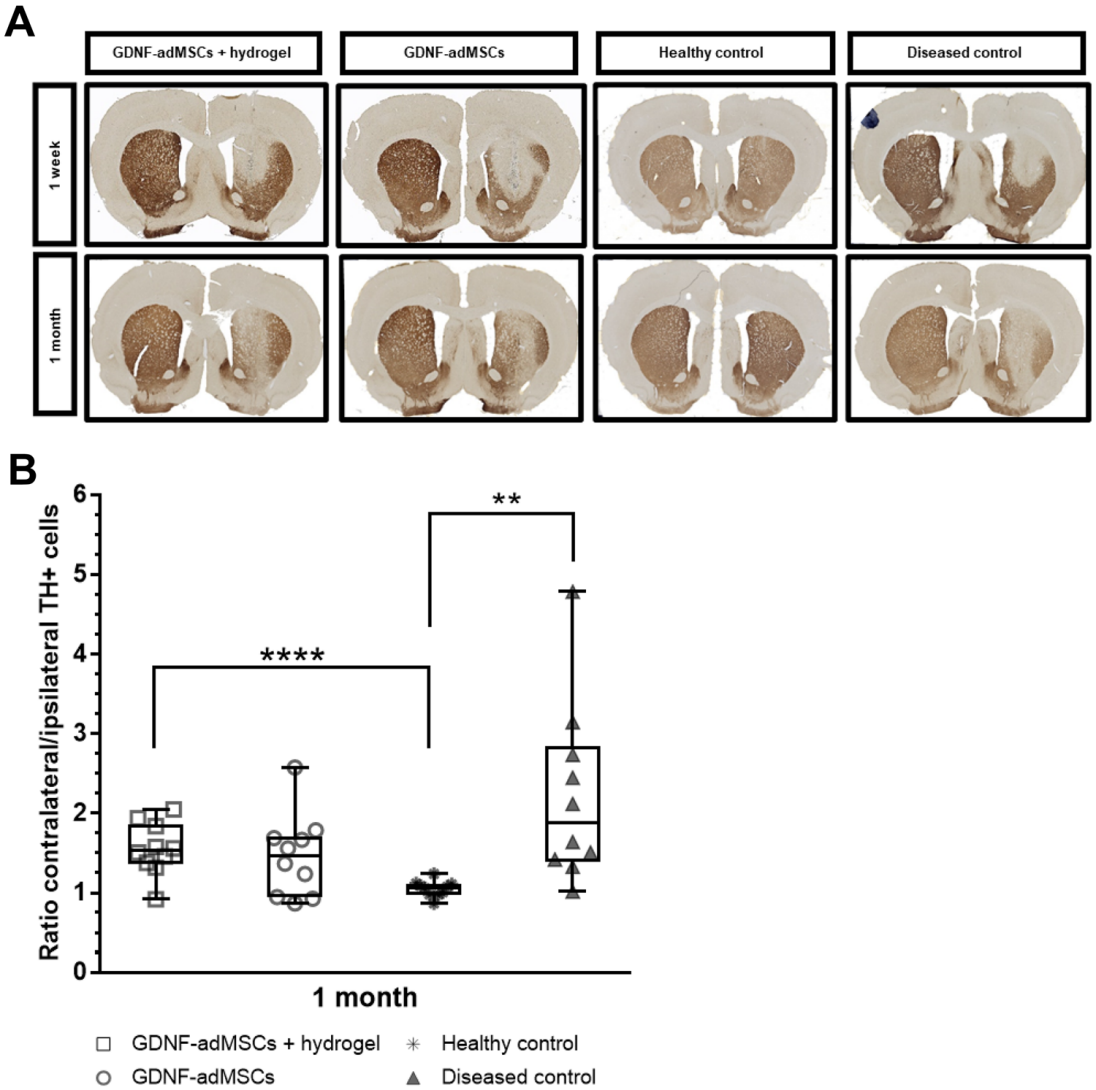
TH expression level evaluated by striatal tyrosine hydroxylase positive (TH +) staining and stereological nigral TH + cell counting ex vivo. **A** The amount of TH + fibers in the ipsilateral hemisphere was decreased in groups with 6-hydroxydopamine (6-OHDA) lesion, 1 week and 1 month after transplantation surgery. The majority of animals in both the GDNF-adMSC groups showed TH + cells in the transplantation site, suggesting that the transplanted cells are expressing TH. In contrast, no TH + cells were noticeable in the control groups. TH + fibers in both striatal hemispheres in the healthy control groups were uniformly visible at the same timepoint. **B** Stereological quantification was used to determine the number of nigral TH + neurons. Data is displayed as the ratio of contralateral (non-operated hemisphere) to ipsilateral (operated hemisphere) cell numbers. Boxes indicate 25^th^ and 75^th^ percentiles and median, and whiskers indicate min and max values; **p < 0.01, ****p < 0.0001

Effect of the different transplants on TH expression levels in striatal CPu (TH + fibers) and SNpc (TH + cells and fibers) was evaluated by immunohistological staining as well as stereological counting for SNpc. As predicted, the healthy control group showed the same high TH + expression pattern in the ipsilateral and contralateral striatum and SNpc. This equal ratio of TH + neurons 1 month after transplantation (median: 1.07, IQR: 0.98 to 1.11) was significantly different to almost all other groups (Fig. 6A, B). Equal striatal TH + fiber staining on both hemispheres in both healthy groups 1 week and 1 month after transplantation is visible as well (Fig. 6A). Compared to the healthy control, the ratio (contralateral/ipsilateral) of TH + neurons in the diseased control was two times higher than that in the healthy control 1 month after transplantation (median: 1.88, IQR: 1.40 to 2.84; healthy control vs. diseased control **p = 0.0019). The progressive neurotoxic effect of 6-OHDA on the dopaminergic TH + neurons is confirmed by the decreasing TH + expression after 1 month compared to 1 week after transplantation (Fig. 6A). Although the entrapped GDNF-adMSC group showed a significantly higher TH + ratio compared to healthy control (median: 1.54, IQR: 1.37 to 1.87; healthy control vs. GDNF-adMSCs + hydrogel ****p = 0.00007), entrapped GDNF-adMSCs led to a considerable but not significant improvement compared with the diseased group. This was also confirmed histologically, as seen by the visible increase in TH + stained fibers 1 month after transplantation, compared to the diseased control group (Fig. 6A). One week after transplantation, the decrease of TH expression in the group with entrapped GDNF-adMSCs was comparable to the diseased control group despite transplantation of entrapped GDNF-adMSCs, indicating a potential time-dependent effect. Treatment of GDNF-adMSCs without hydrogel showed a nonsignificant trend toward healthy control TH + ratio (median: 1.47, IQR: 0.95 to 1.72), and similar to the entrapment GDNF-adMSC group, a trend of an increased amount of TH + fibers was observed compared to diseased control after 1 month (Fig. 6A). Similarly, the amount of TH + neurons in GDNF-adMSCs was increased 1 month after transplantation compared to 1 week post-transplantation. Taken together, the GDNF-adMSC-treated groups showed higher presence of TH + 1 month post-transplantation when compared to the untreated control and at 1 week post-transplantation, although only differences compared to the healthy control were partly significant.

### Time-dependent tendency to an improvement of motor behavior in GDNF-adMSC groups

**Fig. 7:**
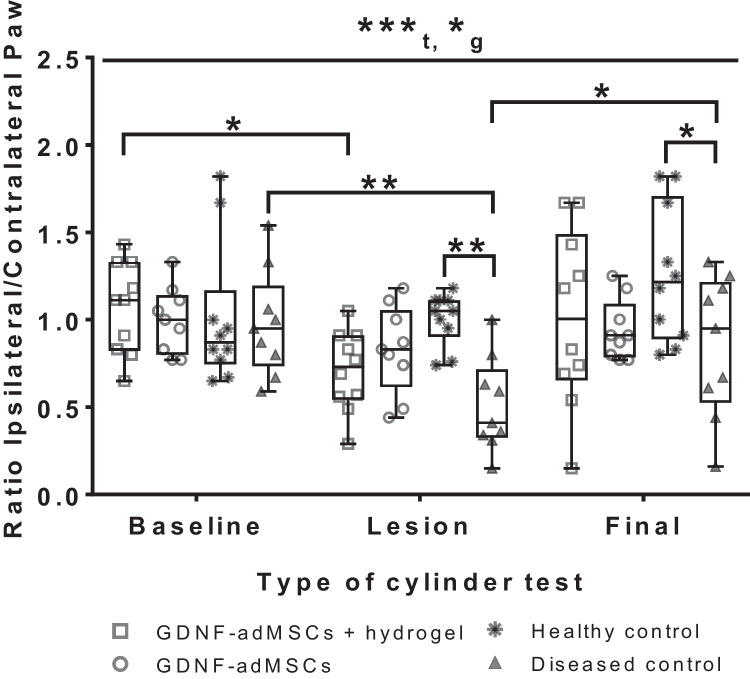
Evaluation of motor behavior via cylinder test. Locomotor asymmetry of rats was measured three times with cylinder tests, starting with a baseline test prior to any intervention, followed by a lesion test 4 days after lesion surgery and ending with a final cylinder test 1 month after transplantation surgery. Wall contacts of the contralateral right paw (impaired paw, controlled by left operated hemisphere) and ipsilateral left paw (non-impaired, controlled by right non-operated hemisphere) were counted and represented as ratios. Boxes indicate 25^th^ and 75^th^ percentiles and median, and whiskers indicate min and max values; *p < 0.05, **p < 0.01. Letters indicate significant factors (*t* = time [***p < 0.001], *g* = group [*p < 0.05]). Grubb’s test showed 1 outlier in the GDNF-adMSC group and 1 outlier in the diseased control group (*n* = 9)

Evaluation of motor behavior of BT animals was measured by paw movement performance in the cylinder test, generating a ratio by comparing the number of contralateral taps (impaired paw, controlled by lesioned hemisphere) with the number of the ipsilateral taps (non-impaired paw, controlled by non-lesioned hemisphere). Healthy animals should show a well-adjusted use of both paws in general, displaying a ratio around 1. Reduced use of the animals’ impaired paws due to 6-OHDA lesions should lead to a ratio less than 1. In general, the timing of the cylinder test (*F* (2, 68) = 9.477, ***t = 0.0002) and the different treatments of the groups (*F* (3, 34) = 3.621, *g = 0.0227) were significant factors and there was no significant interaction between the two factors. As expected, medians of baseline ratios from all groups yield around 1 with no significant differences between the groups (Fig. 7). Time-dependent changes after lesioning with 6-OHDA were measurable, and 6-OHDA lesion led to less usage of the contralateral paws in the GDNF-adMSC-treated groups and the diseased control group. Comparing with corresponding baseline cylinder tests (before lesion surgery), the number of contralateral taps of GDNF-adMSC + hydrogel was significantly reduced by 34% on average (baseline vs. lesion, *p = 0.0198) and of GDNF-adMSCs by only 17%. Expectedly, the healthy control group showed no reduction in the use of contralateral paws due to sham lesion, whereas the diseased control group showed the strongest significant reduction of medial of 57% (baseline vs. lesion, **p = 0.0031) compared with respective baseline results. Within the lesioned groups, the diseased control group showed a strong significant reduction of paw ratio at the lesion test compared with the healthy control (61% reduction of diseased control vs. healthy control, **p = 0.0048). A similar, but less significant behavior pattern was observed at the final tests (22% reduction of diseased control vs. healthy control, *p = 0.0306), yet there was also a significant complete recovery of contralateral paw usage of the diseased control to baseline levels (lesion vs. final, *p = 0.0378). Comparing motor performance ratios of final cylinder tests with ratios of lesion cylinder tests, the experimental groups recovered their ratios to almost baseline results on average, but with no significant treatment effect. However, higher usage of contralateral paws of healthy and diseased control groups in final cylinder tests compared to lesion cylinder tests was also measured, but with highest variances. All in all, starting from the same baseline conditions in all groups, the diseased control showed significantly reduced contralateral paw usage throughout both subsequent cylinder test timepoints. Nonetheless, 6-OHDA lesion did not impact all groups in the same way, and still improvement trends in motor behavior in both GDNF-adMSC groups were noted.

## Discussion

There is still a high and unmet need for the development of neuroprotective, disease-modifying therapies for patients diagnosed with PD. Here, we have demonstrated a potential stem cell therapy in a mild rat 6-OHDA PD model. Human adMSCs were transfected with a SB transposon to overexpress GDNF and then transplanted either alone or entrapped in a fibrin hydrogel. The hydrogel was used as an auxiliary biomaterial to aid cell delivery and boost the putative neurorestorative effects of MSCs and led to anti-inflammatory responses. GDNF synthesis was evident in the diseased tissue of 6-OHDA-lesioned rats, and this GDNF delivery attenuated the loss of TH + neurons.

In the present study, we established a suitable transfection procedure for transfecting adMSCs with a SB transposon system to overexpress GDNF in significantly higher amounts compared to naïve adMSCs. With this approach, strong in vitro gene overexpression was detectable from 24 h to 2 months independent of the selected gene of interest. Several studies have already shown the ameliorative effects of GDNF overexpression in vivo using viral vectors in stem cells, but not via SB transposon in a PD model [39, 45, 58]. SB transposons represent an interesting alternative due to their long list of advantages, including decreased production costs, increased biosafety, and low immunogenicity [59]. Compared to the viral vector systems, only a few SB approaches have reached clinical trials, since there are still several aspects which need to be improved [60–62]. For example, SB transposons have a safer integration profile, but the integration mechanism still follows a random pattern; thus, it is not possible to site-specifically integrate transgenes into the host cell genome. Yet, the targeting of the SB transposons into defined genomic regions has been challenging [36]. In order to overcome this problem, one could combine the SB transposase with directing tools such as components of the CRISPR/Cas9 system. Therefore, either the SB transposase can be fused directly with the DNA-binding domain of the Cas nuclease or via an adapter protein, guiding the SB transposase to a specific target sequence, recognized by the DNA-binding domain [63].

Functional GDNF-adMSCs were successfully transplanted into rats with a mild hemiparkinson lesion model. Using GDNF and NuMa immunostainings as markers, GDNF-adMSCs were still visible and functional according to secretion of GDNF 1 month after transplantation. However, both experimental groups showed the presence of fewer GDNF-adMSCs after 1 month in comparison with 1 week. A possible reason of this decreasing adMSC numbers could be the potential differentiation of GDNF-adMSCs into neural cell-like cells or which scientists have already demonstrated in several in vitro studies [64, 65]. Although potential neurorestorative and neuroprotective effects of overexpressed GDNF can diminish during differentiation, the presence of functional neural cells instead would add the potential for regeneration and therefore improvement of disease-related characteristics, for example by increasing TH + cell numbers. The reason for not using dopaminergic neurons or neural precursors right from the beginning lies in translational hurdles, for example finding convenient and ethical stem cell sources and preventing immune system reactions as much as possible for longer transplant survival. Therefore, MSCs are more suitable, especially adMSCs, due to their high availability and easy extraction with no ethical issues, even enabling autologous cell transplantation. Decreased cell numbers could also be the result of migration of GDNF-adMSCs from the transplantation location or low cell survival. Determining cell viability and vitality in the cell transplant in vivo is challenging. Constitutively expressing reporter genes like GFP could have been used to determine the fate of transplanted cells. In combination with cell death analysis such as terminal deoxynucleotidyl transferase dUTP nick end labeling (TUNEL) assays, potential cell death of transplanted cells and migration would have been traceable. We apprehended poor transduction rates when adding a GFP reporter gene to the vector since our vector was already quite large due to the SB vector design and GDNF gene. However, it is essential to implement further testing for detailed vitality and viability parameters of adMSCs not only after transfection, but also after entrapment in fibrin hydrogel. This is necessary to validate the approach so that optimal transplantation condition of cells is guaranteed.

Although cell numbers decreased in both the GDNF-adMSC group and the GDNF-adMSC group with hydrogel, entrapment of GDNF-adMSCs in hydrogel led to slightly higher cell numbers and longer GDNF secretion, supporting the idea that even a hydrogel with short half-life can promote beneficial cell functions. Several studies evaluated the problem of low cell survival of transplanted cells and identified reasons like immune system reaction or homing [39, 66]. Our method to counteract these factors was the combination of GDNF-adMSCs with a biodegradable fibrin hydrogel. The idea was to promote cell growth and survival by providing an instant hydrogel matrix which can act as a scaffold, also providing a defined niche for the cells to reside in directly after transplantation surgery. Furthermore, this scaffold can minimize cell migration since the cells are kept together for the first days after transplantation in the hydrogel, enabling them to produce and build their own matrix. Nonetheless, a hydrogel with slower degradation time or different approaches like catheter-like devices with perm-selective hollow fibers could possibly lead to higher local cell numbers. Paolone et al. [67] were able to generate GDNF overexpressing ARPE-19 cells with the SB transposon expression system and entrapped those cells in an immunoprotective hollow fiber membrane. They were implanted in epileptic rats and showed substantial GDNF secretion for 8 weeks before concentrations slowly decreased in the devices. These catheter-like devices are especially interesting since they can be retrieved if side effects occur [67].

Supposing that the major components of hydrogel were already degraded after 1 month, the fibrin hydrogel seems to still have delayed beneficial effects on MSCs’ immunomodulatory function. Low levels of IL-1β in the entrapped GDNF-adMSC 1-month group were detected, indicating a potential reduction of pro-inflammatory effects. Although levels of several pro-inflammatory cytokines tend to be nonsignificantly higher in both experimental groups at both timepoints, the adMSC + hydrogel group showed significantly lower levels of pro-inflammatory IL-1β after 1 month compared to the respective group without hydrogel. Surprisingly, all significant differences can only be seen in the 1-month group with hydrogel and no significantly lower levels of IL-1β were measured in the 1-week group with hydrogel. This delayed immunomodulatory effect can be explained by the results of Ren et al. [68], where they discovered that the presence of pro-inflammatory cytokines like TNF-α or IL-1β is initially necessary to activate the immunosuppressive function of MSCs later. The hydrogel may lead to a stronger self-built MSC matrix and higher amounts of cells, which can lead to higher anti-inflammatory effects.

One further observation from this study is the time-dependent changes in TH + cell amounts and motor behaviors, although the differences between different GDNF-adMSC groups were not significant. A potential cause for this could be the relatively short half-life of the chosen fibrin hydrogel. The fibrin hydrogel already starts to degrade after a few days [45], coinciding with our immunostaining (Supplementary Fig. S3) and MRI results (Fig. 3A), where hydrogel size was already reduced after 1 week. This leads to the assumption that synthetic hydrogels with longer half-times may be better candidates for promoting measurable motor improvements. For future studies, a slow-degradable hydrogel needs to be designed to guarantee long-term survival and local restriction of GDNF-adMSCs while still permitting neuroprotective and neurorestorative functions of the entrapped MSCs.

Regardless of whether the cells were entrapped or not, we could demonstrate slightly improved trends of nigral TH + numbers and fibers compared to the control groups. However, we were not able to examine significant motor improvements since the 6-OHDA lesion did not impact all diseased groups in the same way. The GDNF-adMSC-treated groups did not show the same expected severe motor impairment as the diseased control at the lesion timepoint. This difference in lesion severity and also the greater variances at the final cylinder tests in the control groups and GDNF-adMSC + hydrogel group suggest that the mild 6-OHDA model with this limited number of animals was not sufficient to measure significant behavioral differences among the groups in this type of behavior test, but only tendencies. One possible reason for this can be that the used concentration of 6-OHDA was too low to detect motor deficits, although Spinnewyn et al. [69] could detect a significant limb use asymmetry in the cylinder tests and a striatal DA level decrease by 70% with the same total 6-OHDA concentration in all lesioned rats. Different surgery procedures like splitting the total 6-OHDA amount in two injections for a more stable lesion are maybe causative for these differing outcomes [69]. Previous studies are difficult to compare since they all differ in surgery parameters like exact stereotaxic coordinates, time lag between lesion and transplantation surgery, or the way of GDNF application, and every single factor could have an influence. We have to emphasize that this exact setup with this lesion model, these GDNF-adMSCs, and the combination with the fibrin hydrogel is completely new. Consequently, there is no exact reference we can refer to. A major drawback of the 6-OHDA rat model is the acute and severe degeneration of dopaminergic neurons in contrast to the actual progressive degenerations process which takes place in adult PD patients. Additionally, several studies showed that a complete lesion of cells substantially restricts the potential of regenerative therapy approaches [70, 71]. For those reasons, we aimed to use a more translational and therefore mild design of disease model with suitable timing of lesion and treatment. Detrimental to this mild model approach is the risk of having variable reactions of each test animal to the low concentrated neurotoxin, resulting in higher variability than usually occurring in similar models. Although this weakens our analyses, it is maybe closer to variable PD peculiarities in daily medical reality. Due to the heterogeneity of PD, the presented therapy concept would cover different disease stages. And still, it was severe enough to elicit significant TH + cell reduction in the diseased control, leading to the desired partially damaged nigrostriatal system in a retrograde process over a period of a few weeks [72, 73]. One way to address this issue and yet remains at a mild 6-OHDA concentration could be to screen out less-lesioned animals after the lesion cylinder testing. In further studies, a testing of our GDNF-adMSC hydrogel transplant with different striatal 6-OHDA deposit numbers and different 6-OHDA model severeness levels to measure potential GDNF effect on also multiple motoric behavior tests would be interesting.

## Conclusions

In conclusion, GDNF-adMSCs entrapped in hydrogel present a great opportunity to find a potential treatment for PD. The use of the SB transposon system proved to be beneficial for the successful overexpression of GDNF in sufficient amounts and in relevant time periods. The hydrogel appears to support GDNF-adMSCs in their effect and showed promising anti-inflammatory effects; nevertheless, the stability needs to be prolonged to ensure the full potential of the GDNF-adMSCs. Further motor behavioral tests and experiments need to be tested in order to fully evaluate this treatment option.

## Supplementary Information

Below is the link to the electronic supplementary material.

Supplementary file1 (DOCX 6203 KB)

## Data Availability

All data sets are either provided in the figures and supplementary material or are otherwise available upon request.

## Abbreviations

6-OHDA: 6-Hydroxydopamine
adMSCs: Adipose tissue–derived mesenchymal stromal cells
AAV: Adeno-associated virus
BSA: Bovine serum albumin
CPu: Caudoputamen
DAPI: 4′,6-Diamidino-2-phenylindole
eGFP: Enhanced green fluorescent protein
ELISA: Enzyme-linked immunosorbent assay
FDA: Fluorescein diacetate
GDNF: Glial cell-derived neurotrophic factor
IFN-γ: Interferon-γ
IL: Interleukin
IQR: Interquartile range
KC/GRO: Keratinocyte chemoattractant/growth-regulated oncogene
min: minutes
MRI: Magnetic resonance imaging
NuMa: Nuclear mitotic apparatus protein 1
PBS: Phosphate buffered saline
PD: Parkinson’s disease
PI: Propidium iodide
SB: Sleeping Beauty
SN: Substantia nigra
SNpc: Substantia nigra pars compacta
TH: Tyrosine hydroxylase
TH+: Tyrosine hydroxylase positive
TNF-α: Tumor necrosis factor-α
