# Supplementary material for: Sleeping Beauty transposon system for GDNF overexpression of entrapped stem cells in fibrin hydrogel in a rat model of Parkinson’s disease
Source: Drug Deliv Transl Res. 2023 Feb 28;13(6):1745–65. doi: 10.1007/s13346-023-01289-9 (PMC10125957; doi:10.1007/s13346-023-01289-9)
Supplement: Supplementary file 1 — Supplementary file1 (DOCX 6203 KB) [file 13346_2023_1289_MOESM1_ESM.docx]

**Supplementary Data**

**
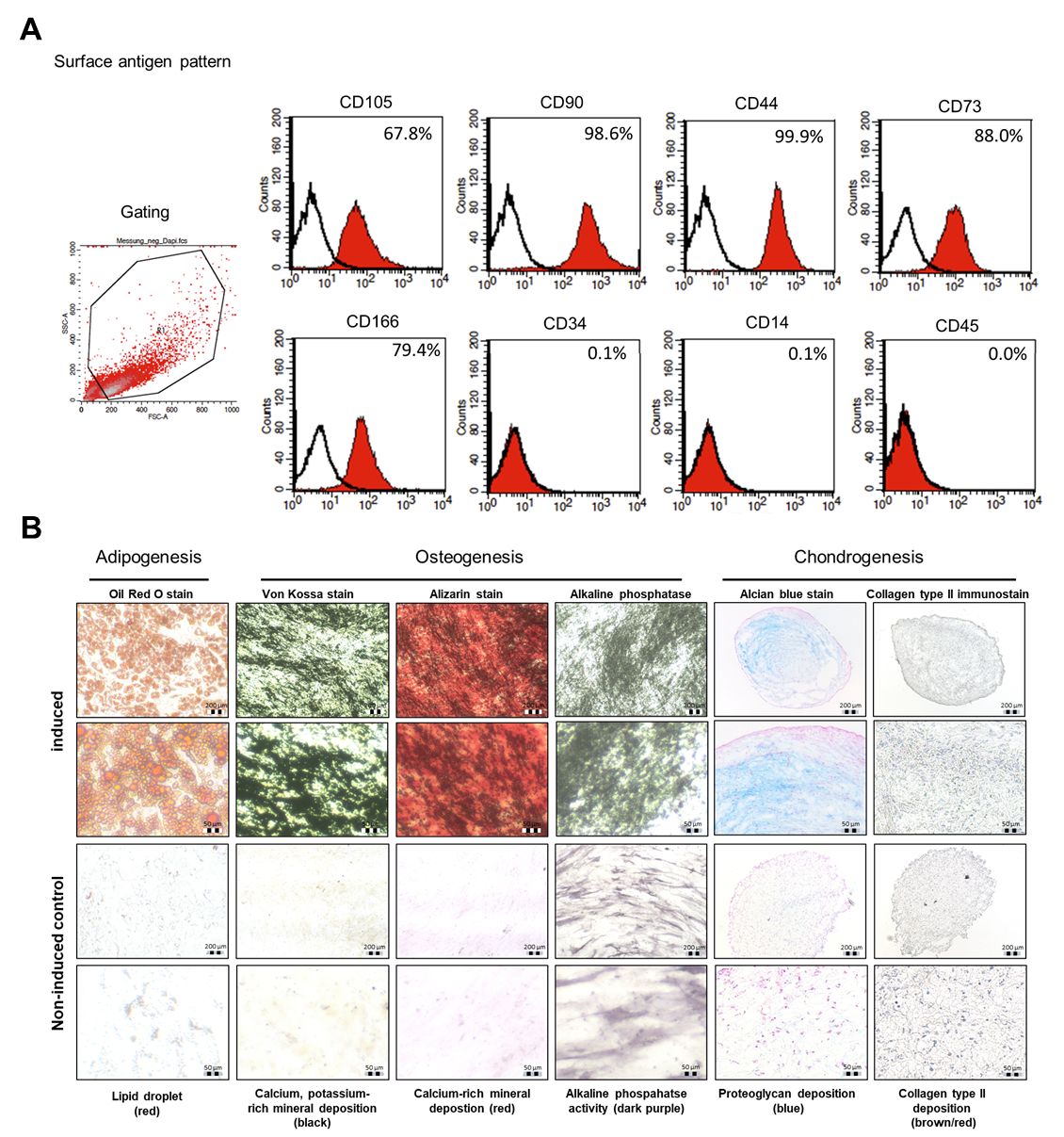
**

**Supplemental Fig. S1: Characterization and differentiation of human adMSCs**. (A) Typical MSC cell surface antigens representation of isolated adMSCs measured by FACS. (B) Multilineage differentiation assays showing adipogenic, osteogenic and chondrogenic differentiation potential.

Abbreviations: FACS: fluorescence-activated cell sorting; adMSCs: adipose tissue-derived mesenchymal stromal cells

**
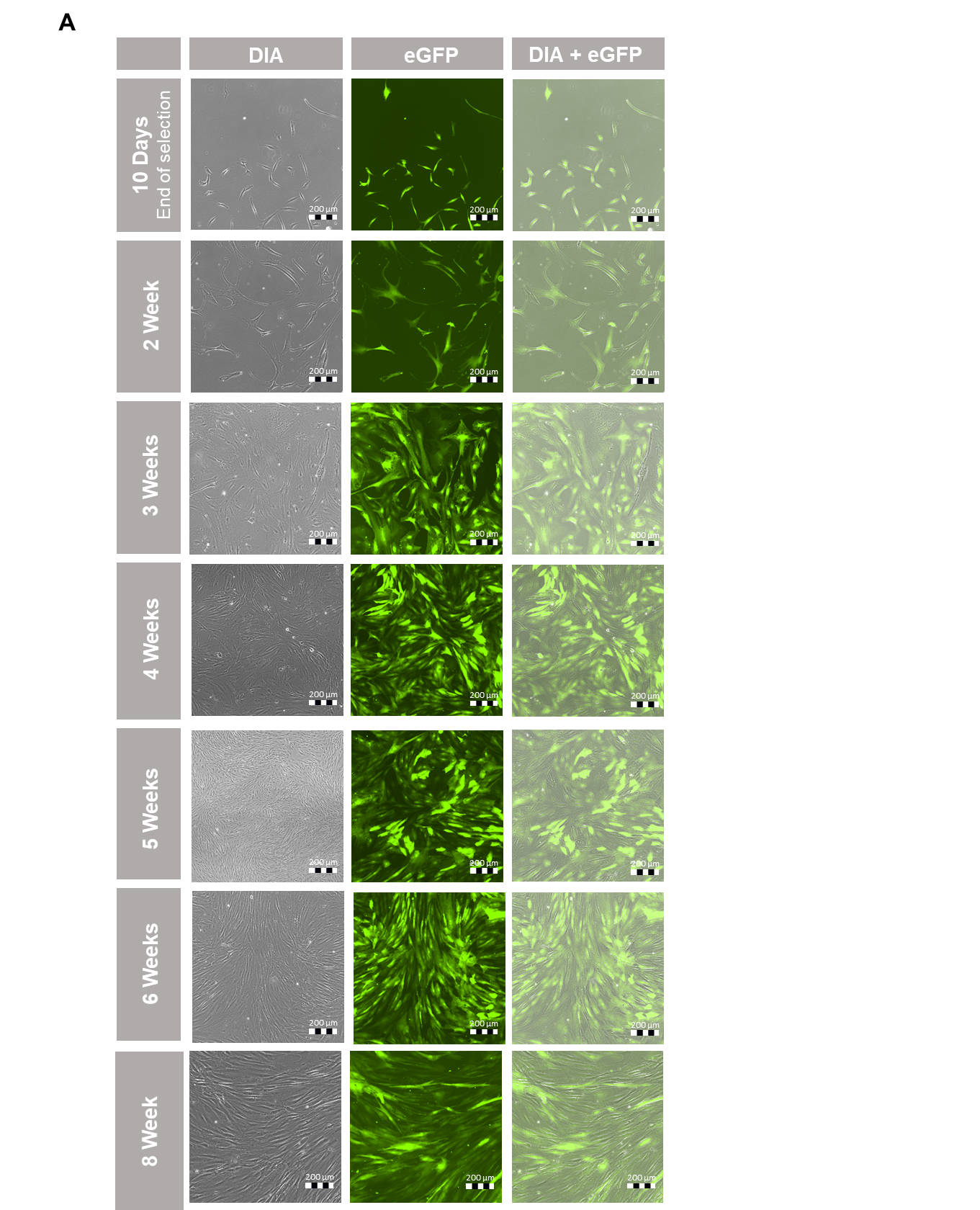
**

**Supplemental figure S2:** Long-term SB-transposon stability and functionality in eGFP-adMSCs. Puromycin-selected eGFP positive adMSCs were detectable up to eight weeks after transfection.

Abbreviations: adMSCs: adipose tissue-derived mesenchymal stromal cells; DIA: transmitted light; GDNF: glial cell line-derived neurotrophic factor; eGFP: enhanced green fluorescent protein; SB: Sleeping Beauty.

**
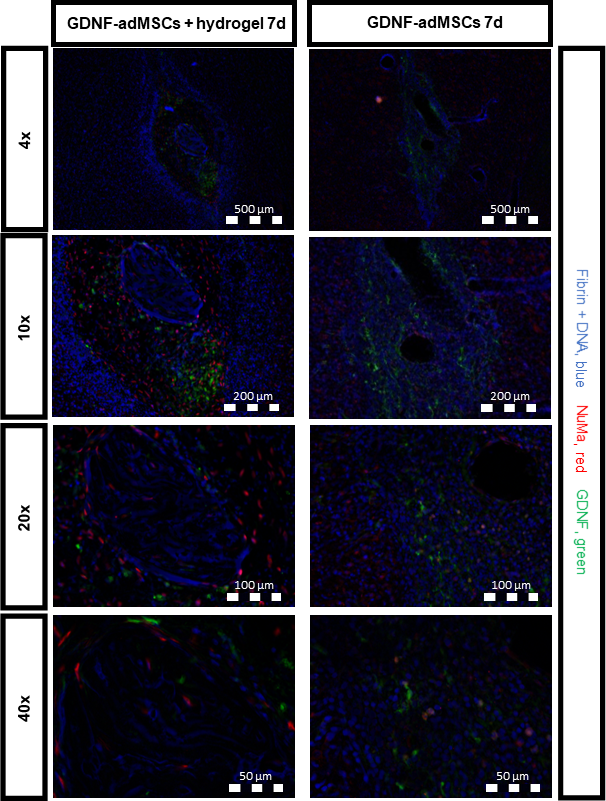
**

**Supplemental Figure S3:** **Visualization of transplant components *in vivo* after one week.** Detection of GDNF-adMSCs (red, NuMa) and secreted GDNF (green) in both experimental groups after 1 week, DAPI (blue) staining for marking cell nucleus and also blue staining for fibrin. Residues of hydrogel are visible within the transplantation cavity of animals in GDNF-adMSCs + hydrogel group, but not in the GDNF-adMSCs group. Only overlays are shown. Higher magnification up to 40x. Abbreviations: adMSCs: adipose tissue-derived mesenchymal stromal cells; DAPI: 4′,6-diamidino-2-phenylindole; GDNF: glial cell line-derived neurotrophic factor; MRI: magnetic resonance imaging; NuMa: Nuclear mitotic apparatus protein 1

**
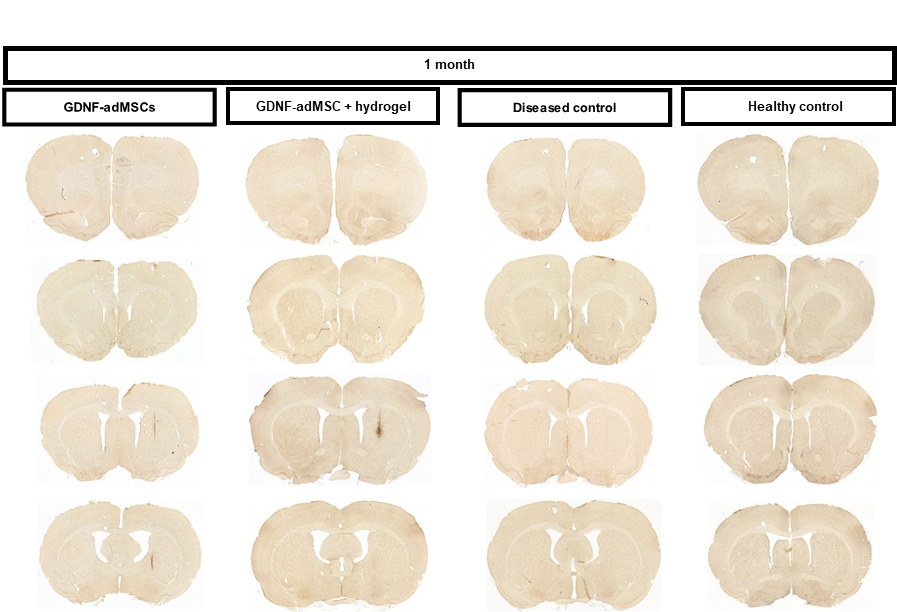
**

**Supplemental figure S4:** ***Ex vivo* GDNF distribution in all groups one month after transplantation.** DAB-GDNF stainings of sequential striatal slices showed low GDNF expression in the GDNF-adMSC group and higher expression levels in the GDNF-adMSC + hydrogel group. No GDNF was detected in healthy or diseased group. Abbreviations: adMSCs: adipose tissue-derived mesenchymal stromal cells; DAB: 3,3'-Diaminobenzidine; GDNF: glial cell line-derived neurotrophic factor


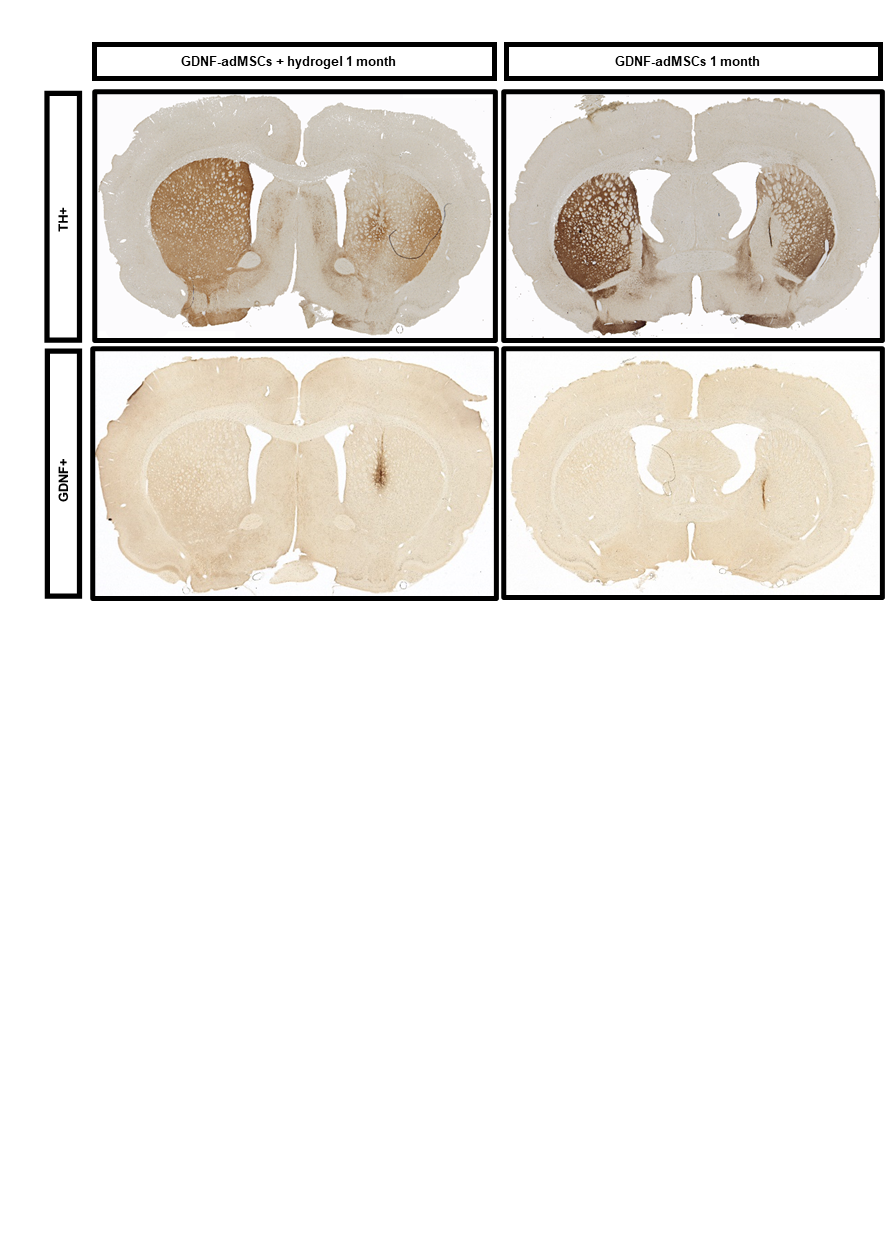


**Supplemental figure S5: Same TH expression and GDNF expression pattern in the transplantation site of GDNF-adMSC transplanted rats in sequential striatal slices *ex vivo*.** In the GDNF-adMSC groups, regions of TH+ and GDNF+ overlap in some animals one month after transplantation of cells, indicating functional expressing tissue. DAB-TH and DAB-GDNF stainings of sequential striatal slices of the same animals.

Abbreviations: 6-OHDA: 6-hydroxydopamine; adMSCs: adipose tissue-derived mesenchymal stromal cells; GDNF: glial cell-derived neurotrophic factor; GDNF+: glial cell-derived neurotrophic factor positive; TH+: tyrosine hydroxylase positive


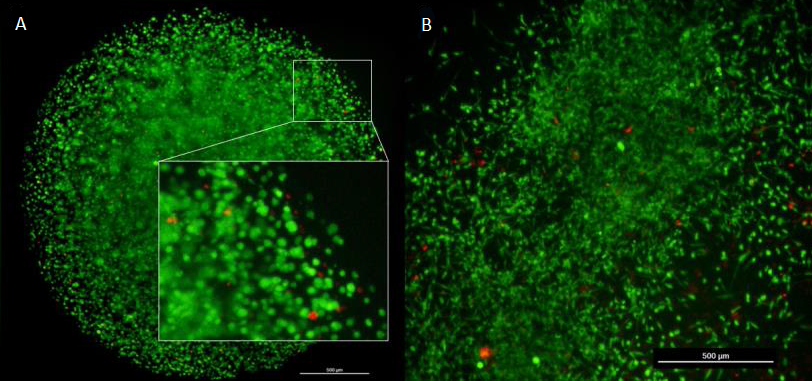


**Supplemental figure S6: Exemplary images of live/dead staining of adMSCs encapsulated in fibrin.** Live cells appear green, dead cells appear red. All pictures taken on day 1 after encapsulation. All encapsulating 20×103 cells. A: Fibrin-NaCl; B: Fibrin-Hyaluronic Acid. In each image, areas showing a representative ratio of live/dead cells are highlighted. Scale bars = 500 μm.


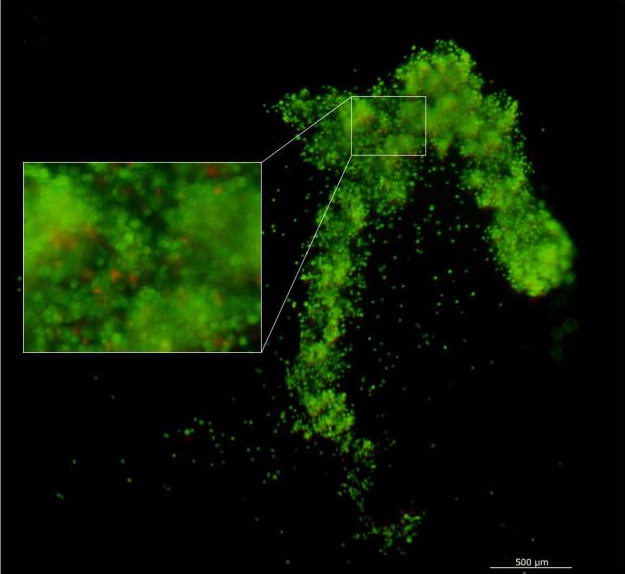


**Supplemental figure S7: Live/dead staining images of adMSCs encapsulated in fibrin via HPLC-syringe immediately after encapsulation (day 0).** Scale bars = 500 μm.
